# Role of glycosylation in bovine leukemia virus infection

**DOI:** 10.1186/1742-4690-8-S1-A29

**Published:** 2011-06-06

**Authors:** Amel-Baya Bouzar, Alix de Brogniez, Arnaud Florins, Carole François, Mathieu Boxus, Luc Willems

**Affiliations:** 1 Cellular and Molecular Biology, Gembloux Agro-Bio Tech, University of Liege, Gembloux, Belgium

## 

As a model for HTLV, reverse genetics can be used in the bovine leukemia virus (BLV) system to identify important mechanisms of viral persistence and pathogenesis. The question addressed here pertains to the role of glycans bound to the BLV envelope glycoprotein (SU gp51). Addition of carbohydrates to the BLV SU potentially creates a structure called « glycan shield » that confers resistance to the virus against the host immune response. On the other hand, glycosylation can also modulate attachment of the virus to the cell membrane.

To unravel the role of SU glycosylation, three complementary strategies were developed: pharmacological inhibition of different glycosylation pathways, interference with glycan attachment and site-directed mutagenesis of N-glycosylation sites in an infectious BLV provirus. Collectively, our results demonstrate that glycosylation is important for the Gp51 maturation process, for virus infection in vitro and for infectivity in vivo.

